# Do fiber tips with different geometric designs affect organic tissue loss in laser-activated irrigation of teeth with immature apex? An in vitro study

**DOI:** 10.1007/s10103-025-04345-7

**Published:** 2025-02-14

**Authors:** Hulde Korucu, Zeliha Uğur Aydın

**Affiliations:** Department of Endodontics, Gulhane Faculty of Dentistry, University of Health Sciences, Ankara, Turkey

**Keywords:** Immature teeth, PIPS, Regenerative endodontics, SWEEPS, Tissue loss

## Abstract

The aim of this study is to quantitatively evaluate the effect of irrigation activation performed with standard needle irrigation (SNI) and laser activated irrigation (LAI) tips of different geometric designs on organic tissue loss in the periapical area of teeth with immature apex. Fifteen single-rooted and canal teeth and seventy-five bovine mucosae were used in this study. An experimental model was constructed, and bovine mucosae were placed in the periapical area. Samples were randomly divided into five groups according to the irrigation activation method (*n* = 15): SNI, PIPS-flat (F), PIPS-radial (R), SWEEPS-flat (F) and SWEEPS-radial (R). Root canals were irrigated with totally 15 mL of 2% NaOCl for three irrigation cycles. Bovine mucosae were weighed before and after the irrigation activation protocols. The difference between the initial and final weights measured organic tissue loss. One-way analysis of variance was performed, followed by post-hoc Tukey significant difference test (*p* < 0.05). The amount of organic tissue loss in PIPS-R was found to be significantly higher compared to PIPS-F (*p* < 0.05). However, there was no significant difference in the amount of periapical organic tissue loss among all other tested irrigation activation methods (*p* > 0.05). All irrigation activation methods caused organic tissue loss. PIPS-R caused more organic tissue loss than PIPS-F, while no difference was found between SWEEPS-F and SWEEPS-R used at the same power setting.

## Introduction

Trauma, caries, and developmental malformations such as dens invaginatus or dens evaginatus may result in pulp necrosis and arrest of root maturation in young permanent teeth [1–3]. Thus, teeth with immature apex, necrotic pulp, and thin and fragile dentin walls are difficult to manage [4]. Due to the thin dentin walls in teeth with immature apex, disinfection is provided by irrigation solutions and intracanal medicaments rather than mechanical instrumentation [5, 6]. Therefore, irrigation activation methods are recommended to increase the effectiveness of irrigation solutions in these teeth [7]. However, irrigation activation methods may cause apical extrusion of irrigation solutions in teeth with immature apex [8]. Apical extrusion of irrigation solutions negatively affects healing and regeneration by damaging periapical tissues and stem cells. Therefore, searching for the ideal irrigation activation method that causes minimal apical extrusion and tissue loss while providing maximum disinfection efficacy in teeth with immature apexes continues [9].

Standard needle irrigation (SNI) is commonly used today due to its ease of application and low cost. However, irrigation solutions show low efficacy in SNI due to limited penetration into the dentinal tubules. In addition, irrigation solutions may extrude apically. Therefore, using different irrigation activation methods is recommended [10].

Another method used to increase the efficacy and distribution of the irrigation solution is laser-activated irrigation (LAI). Er: YAG (2940 nm) lasers are commonly used in LAI due to their suitable wavelength and high absorption in water. In recent years, Photon Induced Photoacoustic Streaming (PIPS) (PIPS^®^, Fotona, Ljubljana, Slovenia, EU), a method of LAI using Er: YAG lasers, emitted in super short pulses (0.3 W, 15 Hz, 50 µs) leading to high power density, very low-temperature evaporation, and low sub ablative power (10–20 mJ) has been developed [11]. PIPS aims to generate cavitation and photoacoustic shock waves in irrigation solutions to create a strong, three-dimensional flow through the root canal system without increasing temperature [11]. For the placement of the PIPS irrigation activation tip, there is less need for root canal enlargement. Therefore, it has been reported that it can effectively deliver irrigation solutions to the apical portion of the root canal system, isthmuses, lateral canals, and resorption areas [12]. The most recent development of LAI in endodontics is the use of Er: YAG laser and Shock Wave Emission Enhanced Photoacoustic Streaming (SWEEPS) (SWEEPS ^®^, Fotona, Ljubljana, Slovenia, EU) using a 600 μm fiber tip inserted into the pulp chamber [13]. The operating principle of SWEEPS is similar to PIPS (0.3 W, 15 Hz, 50 µs, 20 mJ), but the mode of action is different in the SWEEPS technique as synchronized ultra-short pulse pairs are delivered to the solutions. This feature increases shock wave emission even in the narrowest root canals [14]. The amplification of pressure waves produced using SWEEPS has been reported to be greater than the standard PIPS procedure, which emits a single laser pulse [14]. However, fiber tips with different geometries are designed for SWEEPS and PIPS modes. It has been reported that the geometry of the fiber tip affects the bubble shape. The formation of a spherical bubble in radial fiber tips and a channel-like bubble in flat tips has been reported [15]. In the literature, a strong correlation between the geometric design of laser fiber tips and the efficiency of LAI has been reported. Gregorcic et al. [15] emphasized that the design of laser fiber tips directly affects the fluid dynamics within the cavity and the collapse kinetics of cavitation bubbles, determining the intensity and effectiveness of the photoacoustic effects generated during LAI. Specifically, it has been reported that radial fiber tips enhance irrigation efficiency by distributing energy over a wider area along the root canal. In contrast, flat tips deliver localized, high-intensity energy, ensuring effective disinfection of the root canal walls. These findings demonstrate that the geometric design of laser fiber tips plays a critical role in the efficiency of irrigation and the removal of filling materials within the root canal system [16].

In the literature, no study is evaluating the organic tissue loss efficiency of the apically extruded irrigation solution in the periapical area when using SNI and LAI with fiber tips of different geometries in teeth with simulated immature apex. The aim of this study is to quantitatively evaluate the effect of irrigation activation performed with LAI tips of different geometric designs on organic tissue loss in the periapical area of teeth with immature apex. The study’s null hypothesis was that there would be no difference between SNI and LAI final irrigation activation methods with fiber tips with different geometries regarding organic tissue loss in the periapical area in teeth with simulated immature apex.

## Materials and methods

The study design was approved in accordance with the Declaration of Helsinki by the University of Health Sciences Gülhane Scientific Research Ethics Committee (no: 2024-328). The sample size was calculated based on a power analysis using G*Power software 3.1.2 (Universitat, Düsseldorf, Germany) with an alpha error probability of 0.05 and a power of 80% (effect size = 0.25) concerning a recent study of similar design [17]. The power analysis showed that a minimum of 15 samples per group and 75 samples were statistically necessary. For all these reasons, 75 bovine mucosa fragments, and 15 single-rooted, single-canal human mandibular premolar teeth with no internal or external resorption, no coronal caries, and restorations, no cracks and fractures, and no previous root canal treatment, which were extracted for periodontal reasons independently of the study, were included in the study. Since a non-destructive method was to be used, the same 15 teeth were used in all experimental groups, following a recent study of a similar design [17], and the obstacle of anatomical variables was avoided.

Periapical radiographs from the buccolingual and mesiodistal angles confirmed that the teeth met the inclusion criteria, had a single root and canal, and were free of internal/external resorption. The included teeth were soaked in 5.25% sodium hypochlorite (NaOCl) (Cerkamed, Cerkamed Company, Stalowa Wola, Poland) for two days to dissolve organic tissue debris. The tissue residues on the teeth were removed with a periodontal curette. The teeth were stored in 0.1% thymol solution until used in the study.

Access cavities were prepared using a high-speed rotary instrument and a diamond bur. After ensuring apical patency with a #10 K-file (Perfect, Shenzhen, China), the working length was determined to be 1 mm short of the apical foramen. At the specified working length, root canals were prepared to X3 using ProTaper Next (Dentsply Maillefer, Ballaigues, Switzerland). All files were utilized at the torque and rpm values specified by the manufacturer. Following each file change, the root canals were irrigated for 30 s with 2 mL of 2% NaOCl (Cerkamed) using a 30G side-vented needle (Ultradent, South Jordan, UT, USA). To standardize the crown and root lengths, the teeth were marked 5 mm coronally and 11 mm apically from the cementoenamel junction and sectioned at these reference points using diamond disc (Bredent, Senden, Germany), resulting in all samples being standardized to 5 ± 1 mm crown length and 11 ± 1 mm root length. In each sample, an artificial pulp chamber, the reservoir area for irrigation solutions, was prepared in the coronal 5 ± 1 mm section of the canal using a diamond bur [14]. The apical opening was designed to be 1.5 mm in size to simulate an immature apex [18]. For this purpose, Gates Glidden (VDW, Munich, Germany) burs from #1 to #6 (1.5 mm) were used. The root canals were irrigated with 2% NaOCl (Cerkamed), 17% EDTA (Cerkamed), and distilled water, respectively.

**Fig. 1:**
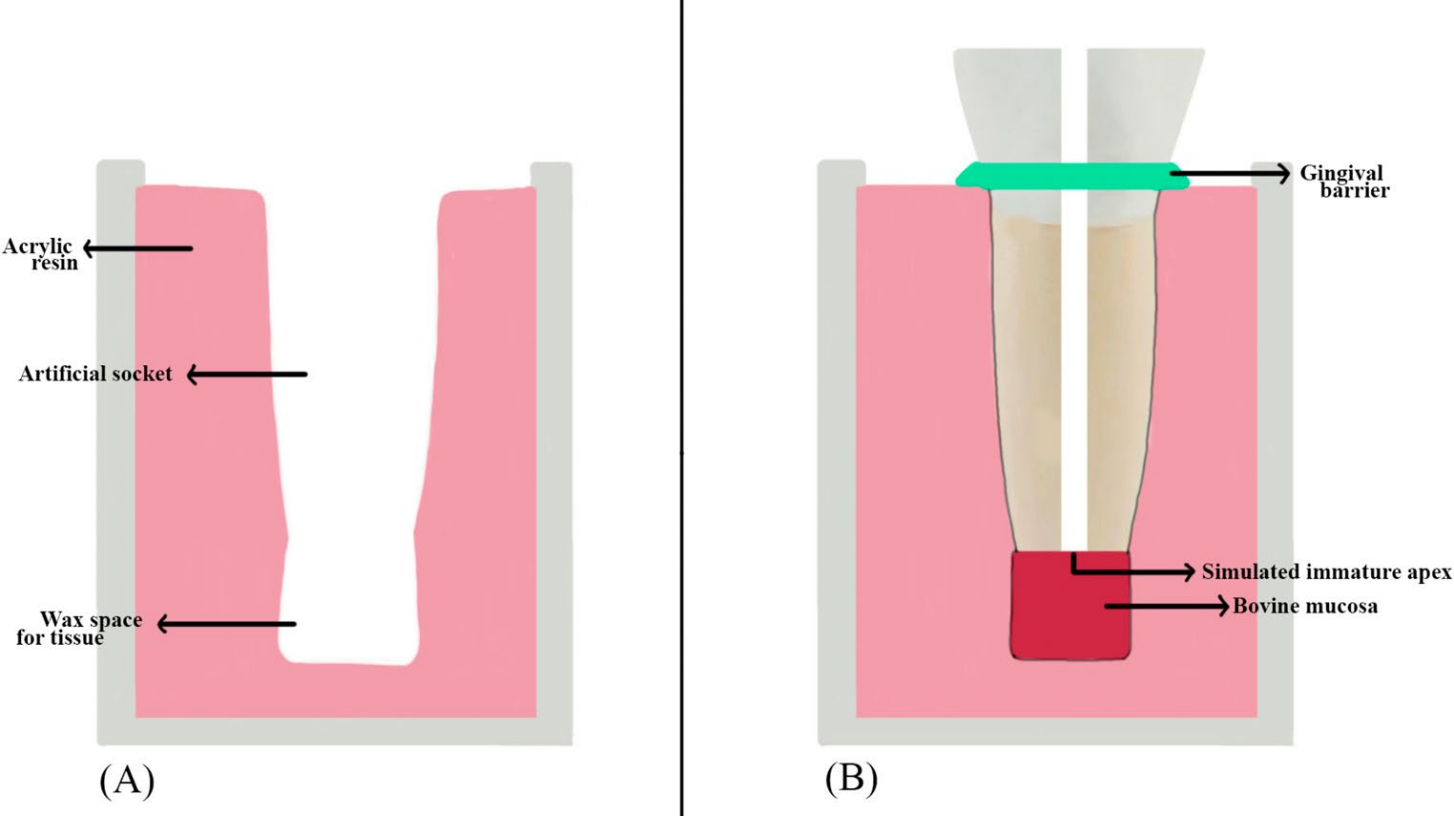
(**A**) An artificial socket model, (**B**) experimental model

The experimental model (Fig. 1) was prepared concerning the method described by Ribeiro et al. [17]. Two layers of overlapping wax sheets with a diameter of 5 mm and a length of 3 mm were placed on the apical part of the teeth and adapted to the tooth with the help of a heated spatula. The tooth’s root was coated with varnish and immersed in acrylic resin. The experimental setup was placed in ice water until the polymerization was complete to prevent the exothermic reaction from melting the wax. The tooth was marked tangentially to the border of the acrylic. After removal from the acrylic container, the apical wax layer was removed. A second mark parallel to the first mark was drawn 2 mm apically. The tissue for simulating periapical tissues was obtained by removing a full-thickness flap from bovine palates obtained from the slaughterhouse and stored in bovine mucosa − 18 °C until use. During the experiment, bovine mucosa with a diameter of 5.5 mm and a height of 5 mm was prepared for each sample and thawed in saline at room temperature for 30 min. All prepared bovine mucosae were weighed on a precision balance (average 70–80 mg). Bovine mucosae were placed in the experimental model. The bovine mucosae were reduced in size with a scalpel until the second parallel line drawn on the tooth was tangential to the acrylic. Bovine mucosae with a distance of less than 2 mm between the first mark drawn on the tooth and the acrylic were removed from the experiment. The bovine mucosae were dried with blotting papers, weighed 3 times on a precision balance, and averaged (mg). This measurement was recorded as the initial weight. The bovine mucosa was placed in the periapical area created in the experimental model, with the epithelium facing the acrylic and the connective tissue facing the root. The tooth was repositioned using a Universal Tester (Instron Corp, Canton, MA) with a compressive force equivalent to 25 gf until the first drawn line was tangent to the acrylic edge. The acrylic and root interface were sealed with a gingival barrier (OpalDam, Ultradent Products, Inc, USA) to maintain a constant back pressure of the tissue on the apex during irrigation. A #100 plugger (Dentsply Maillefer, Ballaigues, Switzerland) was placed up to the apex to compress the portion of bovine mucosae that had entered the canal [17]. The bovine mucosa samples were numbered from 1 to 75. Randomization was performed using computer-generated sequences (www.random.org), resulting in a table that assigned randomized sample numbers to five groups, each containing 15 samples (*n* = 15): Standard needle irrigation (SNI), PIPS-flat (F), PIPS-radial (R), SWEEPS-flat (F) and SWEEPS-radial (R).

### SNI

30G side-vented needle (Ultradent) was placed in the root canal 1 mm shorter than the working length. 5 ml of 2% NaOCl (Cerkamed) was applied for 30 s using 3–4 mm amplitude. Thus, the first activation cycle was completed. The same procedure was applied in the second and third activation cycles. After three activation cycles, final irrigation was performed using 5 ml distilled water.

### PIPS-F

**Fig. 2:**
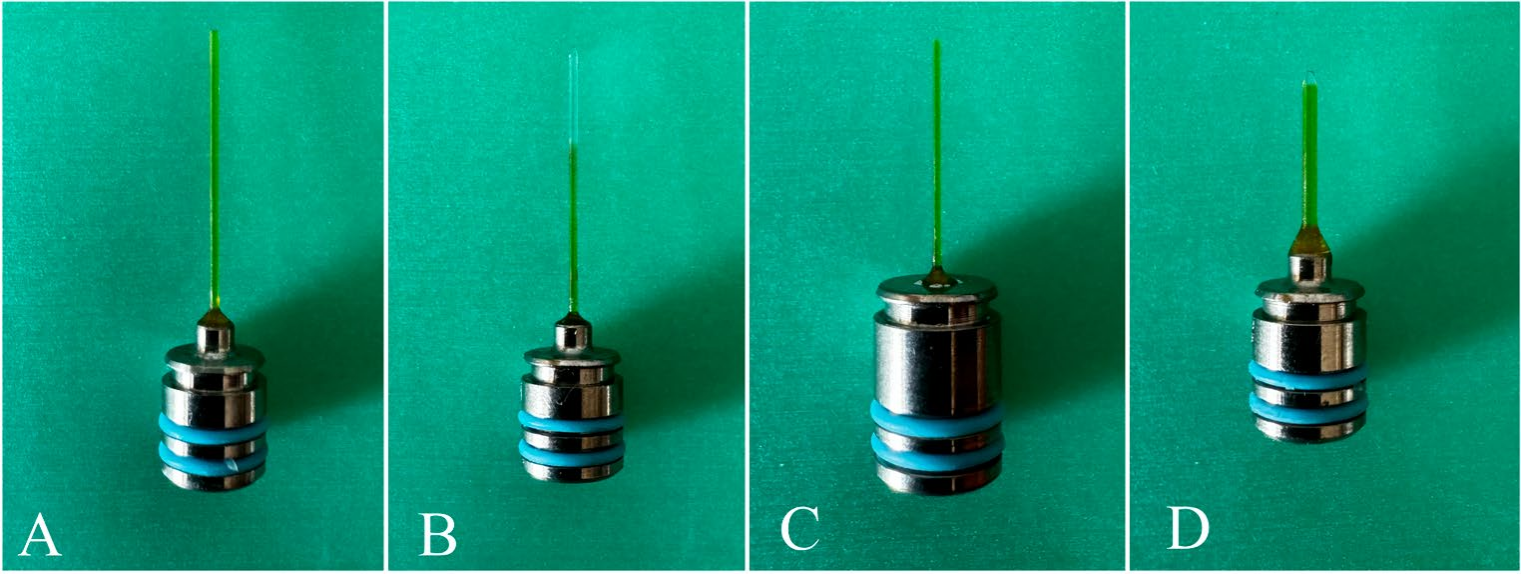
Fiber tip types used in LAI, respectively: PIPS-F (400/14) (A), PIPS-R (400/14) (B), SWEEPS-F (300/9) (C), SWEEPS-R (600/9) (D)

Fotona Light Walker Er: YAG laser was set according to the manufacturer’s instructions at 20 mJ, 15 Hz, 0.3 W in SSP (Super Short Pulse) mode with water and air turned off. PIPS (Fotona, Ljubljana, Slovenia) flat fiber tip (400/14) (Fig. 2, A) was placed in the reservoir area. Three activation cycles of 30 s each were performed using the solutions in the order and volume indicated on the SNI.

### PIPS-R

Without changing the parameters, the PIPS radial fiber tip (400/14) (Fig. 2, B) was inserted into the reservoir field, and the final irrigation activation was performed according to the procedure specified in PIPS F.

### SWEEPS-F

The Fotona Light Walker Er: YAG laser was set according to the manufacturer’s instructions at 20 mJ, 15 Hz, 0.3 W in auto-SWEEPS mode with water and air off. SWEEPS (Fotona, Ljubljana, Slovenia) flat fiber tip (300/9) (Fig. 2, C) was placed in the reservoir area. Three activation cycles of 30 s each were performed using the solutions in the order and volume indicated on the SNI.

### SWEEPS-R

The SWEEPS radial fiber tip (600/9) (Fig. 2, D) was inserted into the reservoir field without changing the parameters. The final irrigation activation was performed according to the procedure specified in SWEEPS-F.

In PIPS-F, PIPS-R, SWEEPS-F, and SWEEPS-R groups, 2% NaOCl (Cerkamed) was continuously added to the reservoir area during activation [19]. After the irrigation activation procedures, bovine mucosae were removed from the experimental model with the help of a fine-tipped tweezer and dried with blotting papers. The dried bovine mucosae were weighed with precision balance three times and averaged (mg). This measurement was recorded as the final weight. The amount (mg) of tissue loss was calculated by subtracting the final measurement from the initial measurement. All procedures were performed by a single endodontist (H.K.).

### Statistical analysis

Shapiro-Wilk test was applied to confirm the normality of the data obtained. Since the data were normally distributed, the weight change was analyzed using One-Way ANOVA and post-hoc Tukey tests. All statistical analyses were performed using SPSS version 23 (IBM, Armonk, NY, USA). *p* < 0.05 was considered statistically significant.

## Results

In the current study, the amount of organic tissue loss in PIPS-R was found to be significantly higher compared to PIPS-F (*p* < 0.05). However, there was no significant difference in the amount of periapical organic tissue loss among all other tested irrigation activation methods (*p* > 0.05) (Table 1).

**Table 1:** Mean weight loss and standard deviation of periapical tissue loss amount for each group

Study group	N/group	Mean of weight loss ± SD
SNI	15	0.020 ± 0.010^ab^
PIPS-F	15	0.016 ± 0.007^a^
PIPS-R	15	0.030 ± 0.017^b^
SWEEPS-F	15	0.017 ± 0.007^ab^
SWEEPS-R	15	0.028 ± 0.015^ab^

SNI, standard needle irrigation, PIPS-F, Photon Induced Photoacoustic Streaming-flat; PIPS-R, Photon Induced Photoacoustic Streaming-radial; SWEEPS-F, Shock Wave Emission Enhanced Photoacoustic Streaming-flat; SWEEPS-R, Shock Wave Emission Enhanced Photoacoustic Streaming-radial.

n: Sample size ±SD: Standard deviation

*P value: Significance level was determined as p < 0.05

## Discussion

In teeth with immature apex, the thin dentin walls limit the application of mechanical preparation [7, 20]. Therefore, disinfection of the root canal system in these teeth depends more on irrigation and intracanal medicament application [6]. For this purpose, using different irrigation activation methods to increase the effectiveness of irrigation solutions is recommended in the literature. However, in teeth with immature apex, the wide, apical area is a potential risk factor for the apical extrusion of irrigation solutions. It has been reported that solutions extruded from the apical area cause pain, burning, and periapical tissue damage. For these reasons, the search for the ideal disinfection method to minimize the extrusion of irrigation solutions continues [21]. In this study, we investigated the effect of SNI and LAI with different tip geometries (flat and radial) on the amount of sodium hypochlorite-induced organic tissue loss extruded apically in teeth with simulated immature apex. The null hypothesis of this study is rejected because there is a difference between the irrigation activation methods tested in terms of the amount of organic tissue loss in the periapical area.

Standardizing variables other than those tested in in vitro studies is essential for an accurate evaluation. In the literature, it has been reported that using the same group of teeth and having similar apical diameter and root canal size to ensure standardization in vitro studies on teeth with immature apex planned to undergo regenerative endodontic procedures [22]. In the present study, mandibular premolars with similar root-to-crown ratios and similar anatomy were used to simulate teeth with immature apices. To achieve dimensional standardization of the teeth, a certain amount of dental tissue was removed from the crowns and apical roots of all teeth, and in the procedure described by Zhabuawala et al. [18], the apical diameter was standardized. In the literature, studies evaluating apical extrusion from different perspectives, such as irrigant and organic loss, have noted that variations in root canal anatomy can lead to potential biases. Many studies have adopted a methodology using the same teeth across all experimental groups to prevent such biases [17, 23]. Similarly, our study employed this approach to minimize the effects of anatomical variations. However, using the same teeth in different groups may lead to changes in dentin buffering capacity. This issue arises because dentin’s potential to interact with irrigants and its buffering properties may be influenced by prior treatments, representing a limitation in our study. Therefore, future research should aim to develop alternative methods that maintain anatomical consistency while eliminating the potential effects on dentin buffering capacity. Similarly, it has been reported that the concentration of the irrigation solution used, the volume of the solution, and the activation time are among the critical variables that affect the results of endodontic treatment [24]. Therefore, all activation methods tested in this study used the same volume of solution with the same concentration, and activation was performed simultaneously. However, in LAI, as opposed to SNI, the volume of solution in the reservoir is carried to the external environment by the effect of the laser, so the continuous solution was added to the reservoir area, similar to previous studies in the literature [19].

The ethical concerns surrounding the use of human palatal mucosa in research have necessitated the evaluation of alternative biological materials. In this context, despite differences in tissue properties as well as physical and chemical structures, bovine palatal mucosa was selected for this study. The structural similarities and ease of availability of bovine mucosa make it a scientifically appropriate and practical option as an experimental model [25, 26].

It was determined that none of the irrigation activation methods tested in the study could prevent the extrusion of the irrigation solution from the apical area. The present study found no difference between SNI and PIPS regarding organic tissue loss in the periapical area. In the literature, no study compares the effect of SNI and LAI performed with different geometry tips on the amount of organic tissue loss in the periapical area. Therefore, the findings of this study were compared with studies evaluating the effect of different irrigation activation methods on irrigation solution and debris extrusion. In contrast to the findings of our study, Azim et al. [27] reported that PIPS caused more solution extrusion than SNI in an experimental root socket model in single-rooted mature teeth. Although the activation time was the same, this difference may be due to the concentration (3%), volume (3 mL), experimental setup, and evaluation methods. Similar to the present study’s findings, Arslan et al. [28] reported no difference between SNI and PIPS with different power settings (0.3–0.9 W) in a modified model using single-rooted mature mandibular premolar teeth. Ince Yusufoglu et al. [29] reported no difference in debris extrusion between SNI and PIPS with a power setting similar to our study (0.3 W) in molars with moderate curvature. In PIPS, cavitation and photoacoustic shock waves are generated in the irrigation solutions, resulting in a powerful three-dimensional flow through the root canal system without temperature increase [11]. The effect of this solid three-dimensional flow in the irrigation solution may have played a role in the fact that SNI and PIPS caused similar solution extrusion in our study.

The present study found no difference between SNI and SWEEPS regarding the amount of organic tissue loss. In contrast to the findings of our study, Vatanpour et al. [30] reported that SNI caused more solution extrusion in immature molars than SWEEPS used at a power setting similar to our study (0.3 W). Abat et al. [31] reported that SWEEPS used at a power setting similar to our study (0.3 W) caused a more significant amount of solution extrusion compared to SNI in a regenerative endodontic procedure by applying 1% 20 mL sodium hypochlorite in three-dimensional immature tooth models with an apical opening of 1.5 mm. These differences may be due to methodological variables such as the morphology of the teeth used in the studies, preparation size, concentration, and volume of solutions. In agreement with the findings of our study, in contrast to these findings, Genç Şen et al. [32] reported no difference in solution extrusion between SNI and SWEEPS in single-rooted teeth of working length and over instrumentation. Since SWEEPS sends synchronized ultrashort pulse pairs to solutions, it increases shock wave emission even in the narrowest root canals [14]. Increased shock wave emission may have played a role in the extrusion of a similar solution caused by SNI and SWEEPS.

In our study, there was no difference in the amount of sodium hypochlorite-induced organic tissue loss extruded apically between PIPS and SWEEPS using radial and flat tips. In contrast to these findings, Snjaric et al. [33] reported that PIPS caused less solution extrusion than SWEEPS after 60 s LAI using 3% sodium hypochlorite in single-rooted mature teeth. This may be due to differences in the concentration of irrigation solution used in the study, tooth morphologies, and laser application time. Consistent with the findings of the present study, Bolhari et al. [34] reported no difference between PIPS and SWEEPS in terms of the amount of methylene blue extruded apically after photodynamic therapy by applying methylene blue in single-rooted mature premolars. No study evaluating the effect of tip design on the organic tissue dissolving efficiency of PIPS and SWEEPS was found in the literature. Although the pulse modes of PIPS and SWEEPS are different, the fact that the energy used, power setting, solution volume, and activation time are the same may have played an essential role in obtaining this result.

Although there was no difference between SWEEPS-F and SWEEPS-R in the present study, it was found that PIPS-R caused more organic tissue loss than PIPS-F. Gregorcic et al. [15] studied the dynamic effect of fiber tip geometries of Er: YAG lasers on liquids and reported that a spherical bubble was formed at radial fiber tips. In contrast, a channel-like bubble was formed at flat fiber tips. He also stated that the total mechanical energy of the liquids is equal to the power in the initial expansion of the bubbles. This energy is more significant when radial fiber ends are used and smaller when flat fiber ends are used. However, as the tip diameters increase, the energy of the bubble formed increases [35]. Therefore, the difference between PIPS-F and PIPS-R may be due to the shape of the bubble formed in the solution.

This study demonstrates that SNI and LAI final irrigation activation methods cannot effectively prevent the apical extrusion of irrigation solutions in teeth with immature apices, potentially resulting in complications such as periapical tissue irritation. The findings reveal that PIPS-R tips cause more significant tissue damage than PIPS-F tips, while SWEEPS produces consistent outcomes irrespective of tip design. Based on these results, it is recommended that irrigation activation methods be applied with precision and control in treating teeth with immature apex, emerging technologies be critically evaluated, and approaches to minimize potential complications be adopted.

This study has certain limitations. Foremost among these is the in vitro design, which does not adequately replicate clinical factors such as tissue healing, immune responses, and the long-term effects of irrigant extrusion on surrounding tissues. The use of bovine palatal mucosa instead of human palatal mucosa introduces potential differences in physical, biological, and histological properties that may influence tissue response. Furthermore, while facilitating the standardization of anatomical variables, the exclusive use of mandibular premolars may have inadvertently overlooked anatomical variations specific to other tooth types. The consistent use of the same teeth in the study may also introduce bias due to the buffering capacity of dentin. To overcome these limitations, advanced studies are needed that better mimic clinical conditions, evaluate a broader range of tooth types, and assess various irrigation activation methods.

## Conclusions

Future research should focus on evaluating the long-term clinical effects of different irrigation activation methods on periapical tissue healing and regeneration. Studies comparing the use of various lasers at different power settings and fiber tips of varying diameters and lengths under clinical conditions are essential to obtain a more comprehensive understanding of the efficacy and safety of these methods. Additionally, exploring alternative disinfection techniques to reduce apical irrigant extrusion could enhance treatment outcomes. Expanding research to include diverse tooth types and patient-specific variables, such as wound healing potential, would further enable these findings to address a broader range of clinical contexts.

## Data Availability

No datasets were generated or analysed during the current study.
